# Supplementary figures and images for: Xrp1 and Irbp18 trigger a feed-forward loop of proteotoxic stress to induce the loser status
Source: PLoS Genet. 2021 Dec 16;17(12):e1009946. doi: 10.1371/journal.pgen.1009946 (PMC8675655; doi:10.1371/journal.pgen.1009946)

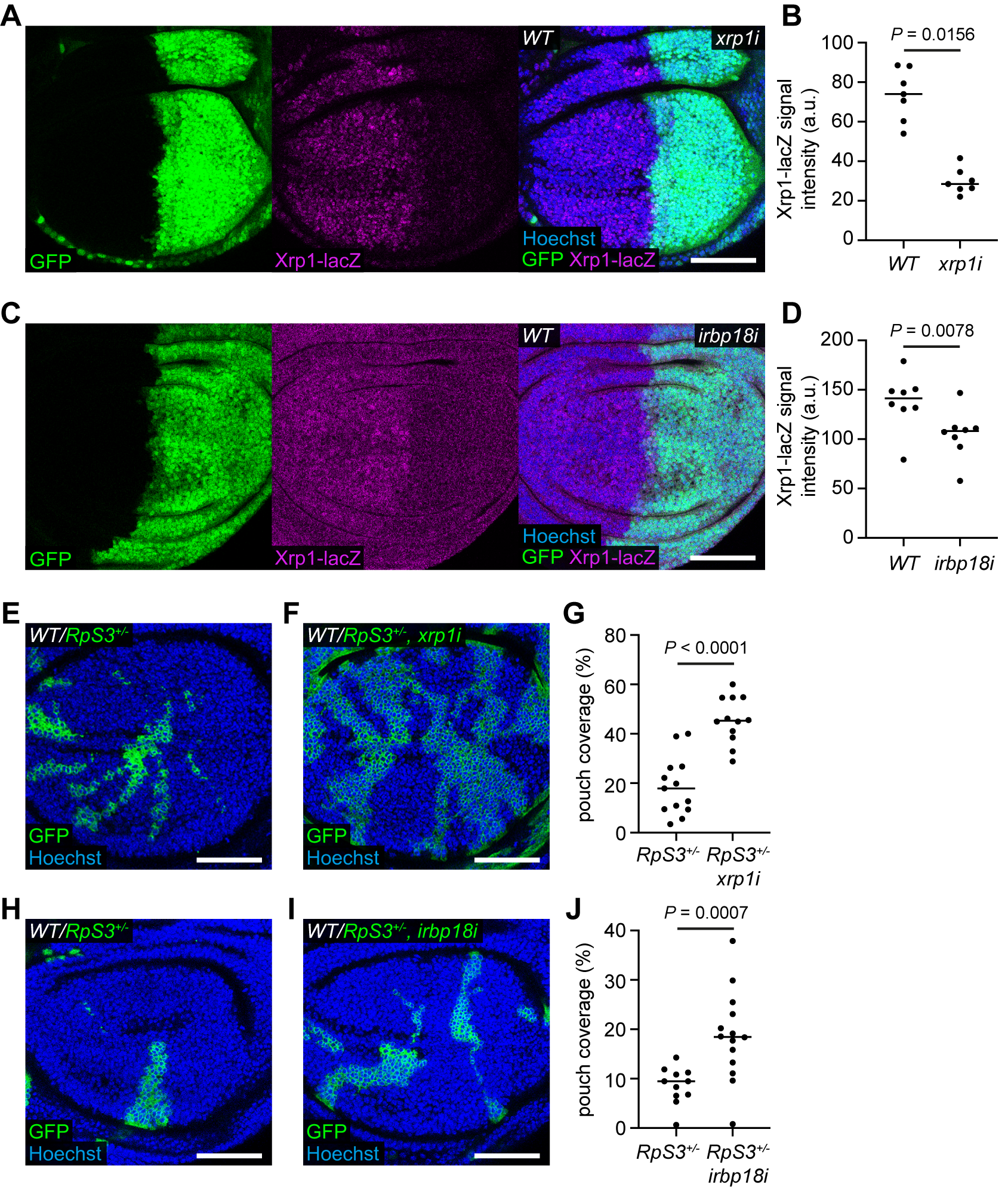

Supplement: S1 Fig — (A-B) A wing disc carrying the xrp1-lacZ reporter and expressing xrp1-RNAi (xrp1i) and GFP (green) in the posterior compartment, immuno-stained with anti-β-galactosidase (magenta) and nuclei labelled with in blue (A), with quantification of xrp1-lacZ signal intensity (B) (n = 7; two-sided Wilcoxon signed-rank test). (C-D) A wing disc carrying the xrp1-lacZ reporter and expressing irbp18-RNAi (irbp18i) and GFP (green) in the posterior compartment, immuno-stained with anti-β-galactosidase (magenta) and nuclei labelled in blue (C), with quantification of xrp1-lacZ signal intensity (D) (n = 8; two-sided Wilcoxon signed-rank test). (E-G) Wild-type wing discs harboring RpS3+/- cells (GFP positive) (E) or RpS3+/- cells also expressing xrp1-RNAi (GFP positive) (F) with nuclei labelled in blue, and quantification of percentage coverage of the pouch (G) (n = 13 and 12, respectively; two-sided Mann–Whitney U-test). (H-J) Wild-type wing discs harboring RpS3+/- cells (GFP positive) (H) or RpS3+/- cells also expressing irbp18-RNAi (GFP positive) (I) with nuclei labelled in blue, and quantification of percentage coverage of the pouch (J) (n = 11 and 14, respectively; two-sided Mann–Whitney U-test). (TIF) [file pgen.1009946.s001.tif]

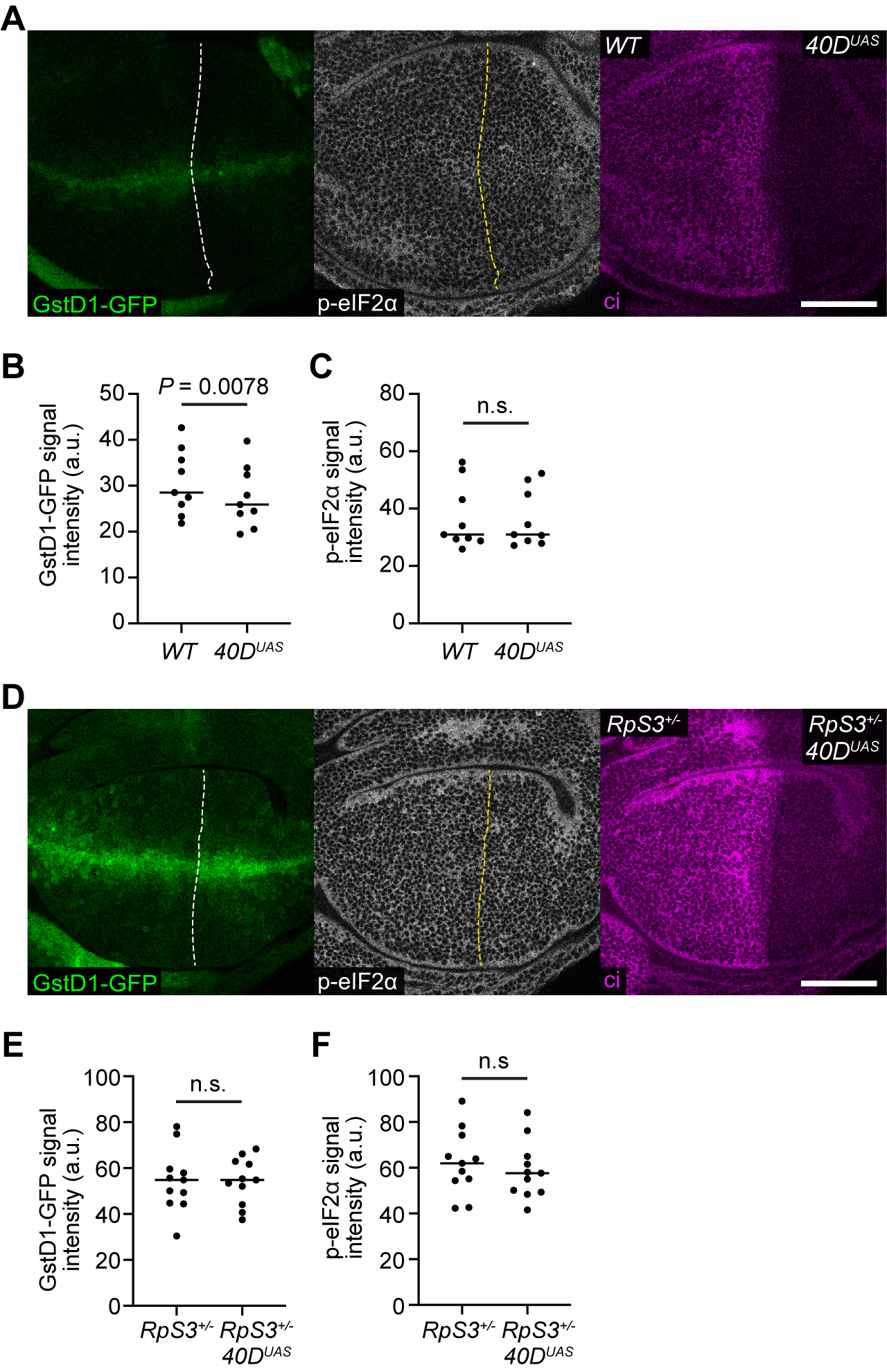

Supplement: S2 Fig — (A-C) A wild type wing disc carrying GstD1-GFP, a posterior Gal4 driver (hh(hedgehog)-gal4), and the 40DUAS insertion used as a control for Gal4 titration. 40DUAS did not markedly affect GstD1-GFP (green) or p-eIF2α (grey) (A). Cubitus interruptus (ci) (magenta) labels the anterior compartment. Quantification of GstD1-GFP (n = 9; two-sided Wilcoxon signed-rank test) and p-eIF2α (n = 9; two-sided Wilcoxon signed-rank test) signal intensity is shown in (B) and (C) respectively. (D-F) An RpS3+/- wing disc carrying GstD1-GFP, hh-gal4, and the 40DUAS insertion. 40DUAS did not affect GstD1-GFP (green) or p-eIF2α (grey) (D). Cubitus interruptus (ci) (magenta) labels the anterior compartment. Quantification of GstD1-GFP (n = 11; two-sided Wilcoxon signed-rank test) and p-eIF2α (n = 11; two-sided Wilcoxon signed-rank test) signal intensity is shown in (E) and (F) respectively. (TIF) [file pgen.1009946.s002.tif]

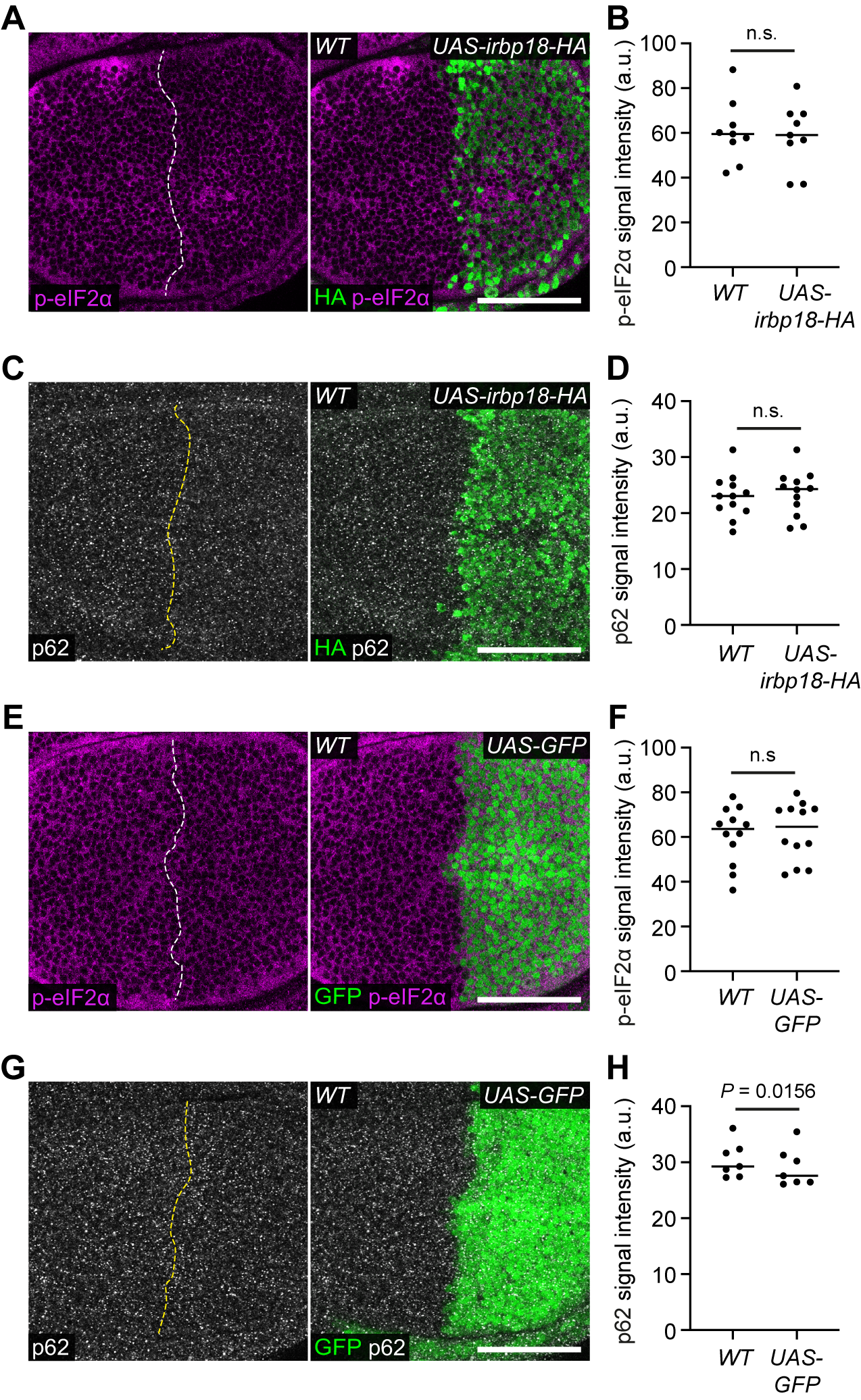

Supplement: S3 Fig — (A-D) Wild-type wing discs over-expressing hemagglutinin (HA)-tagged Irbp18 (UAS-irbp18-HA) in the posterior compartment, immuno-stained for HA (green) and p-eIF2α (magenta) (A) or HA (green) and p62 (grey) (C) with quantification of p-eIF2α signal intensity (B) (n = 9; two-sided Wilcoxon signed-rank test) and p62 signal intensity (D) (n = 12; two-sided Wilcoxon signed-rank test). (E-H) Wild-type wing discs over-expressing GFP (green) in the posterior compartment and immuno-stained for p-eIF2α (magenta) (E) or p62 (grey) (G) with quantification of p-eIF2α signal intensity (F) (n = 12; two-sided Wilcoxon signed-rank test) and p62 signal intensity (H) (n = 7; two-sided Wilcoxon signed-rank test). (TIF) [file pgen.1009946.s003.tif]

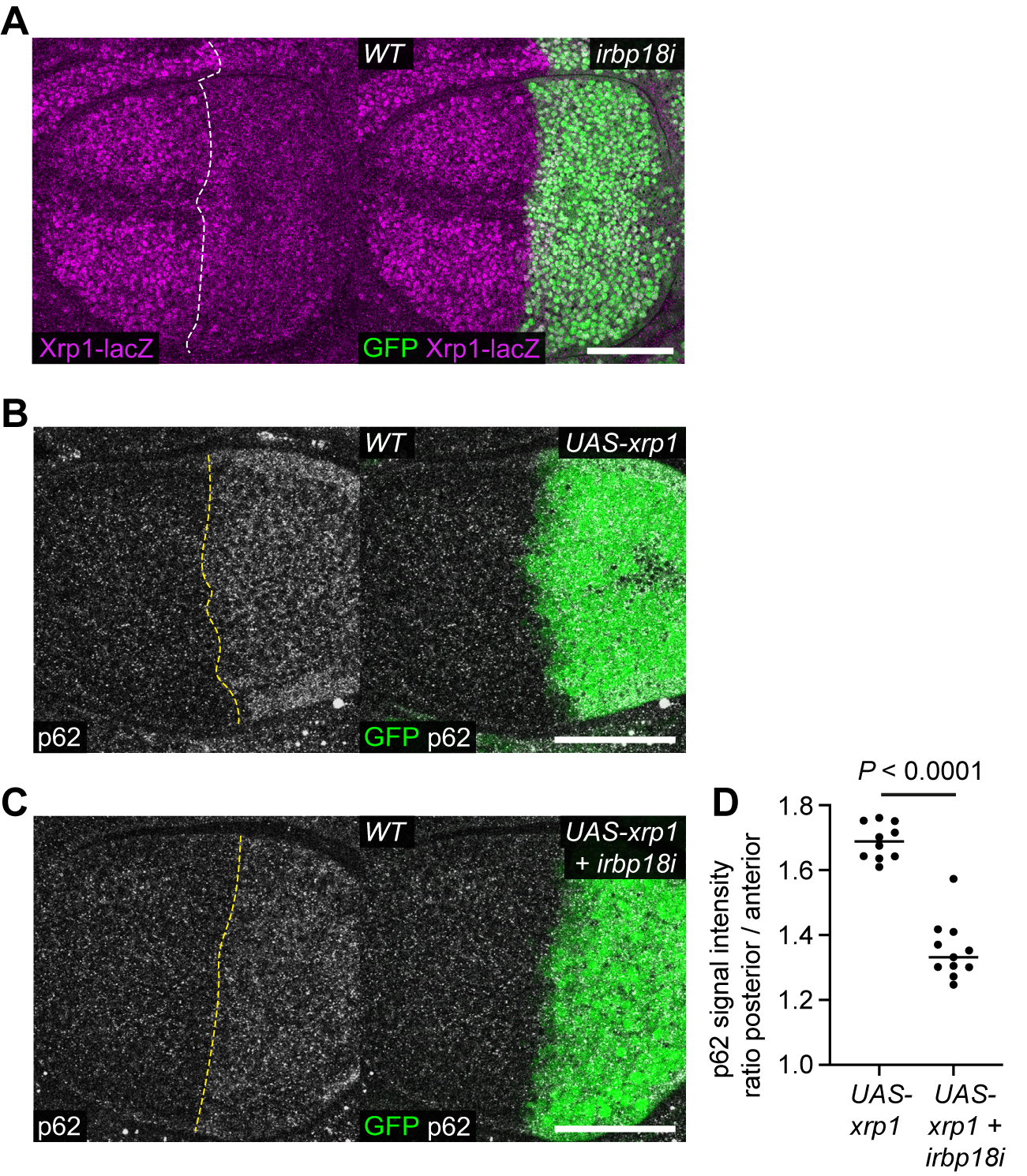

Supplement: S4 Fig — (A) Xrp1-lacZ expression in a wild-type wing disc that has been expressing UAS-ibrp18-RNAi in the posterior compartment (GFP positive) for 24h, as controlled with Gal80ts, immuno-stained for anti-β-galactosidase (magenta). (B-D) Wild-type wing discs that have been expressing UAS-xrp1 (B) or UAS-xrp1 and UAS-irbp18-RNAi (C) in the posterior compartment (GFP positive) for 24h, immuno-stained for p62 (grey) with quantification of the posterior / anterior ratio of p62 signal intensity (D) (n = 10 and 11, respectively; two-sided Mann–Whitney U-test). (TIF) [file pgen.1009946.s004.tif]

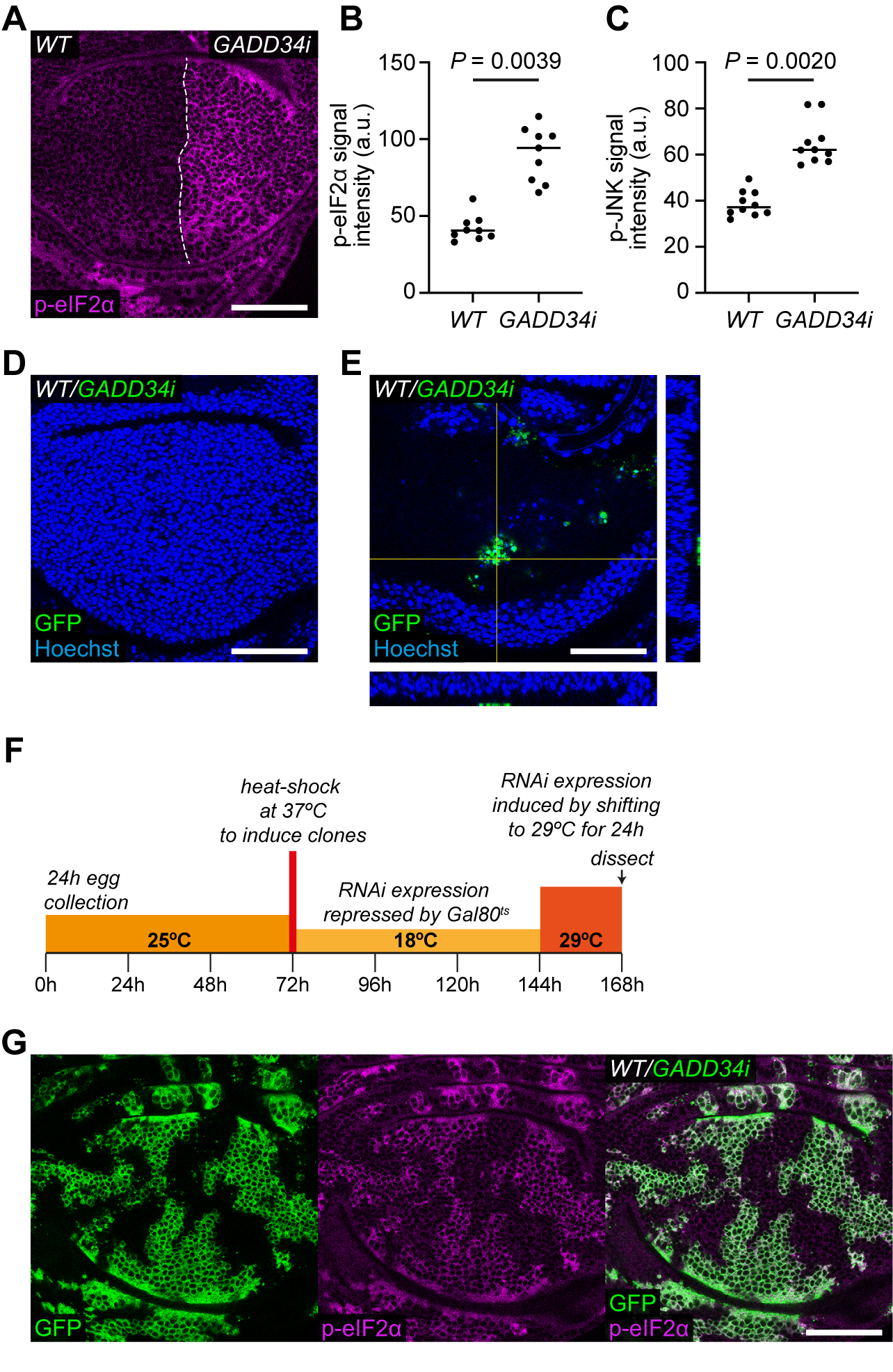

Supplement: S5 Fig — (A-B) A wing disc expressing GADD34-RNAi (GADD34i) in the posterior compartment and immuno-stained for p-eIF2α (magenta) (A) with quantification of p-eIF2α signal intensity (B) (n = 9; two-sided Wilcoxon signed-rank test). (C) Quantification of p-JNK signal intensity in wing discs expressing GADD34-RNAi in the posterior compartment (n = 10; two-sided Wilcoxon signed-rank test). (D) A wing disc harboring GADD34-RNAi expressing cells (GFP positive), generated in the absence of Gal80ts, with nuclei labelled in blue. (E) A basal section of a wing disc harbouring GADD34-RNAi cells (GFP positive), generated in the absence of Gal80ts, with nuclei labelled in blue, to show that only small, basally extruded patches of GADD34-RNAi expressing cells remain. Orthogonal views taken at the positions indicated by the yellow lines are shown to the right and bottom of the main image. (F) Schematic depicting experimental conditions for generating large GADD34-RNAi expressing patches of cells. (G) A wing disc with GADD34-RNAi expressing cells (GFP positive), generated with the experimental conditions depicted in (F), immuno-stained for p-eIF2α (magenta). (TIF) [file pgen.1009946.s005.tif]

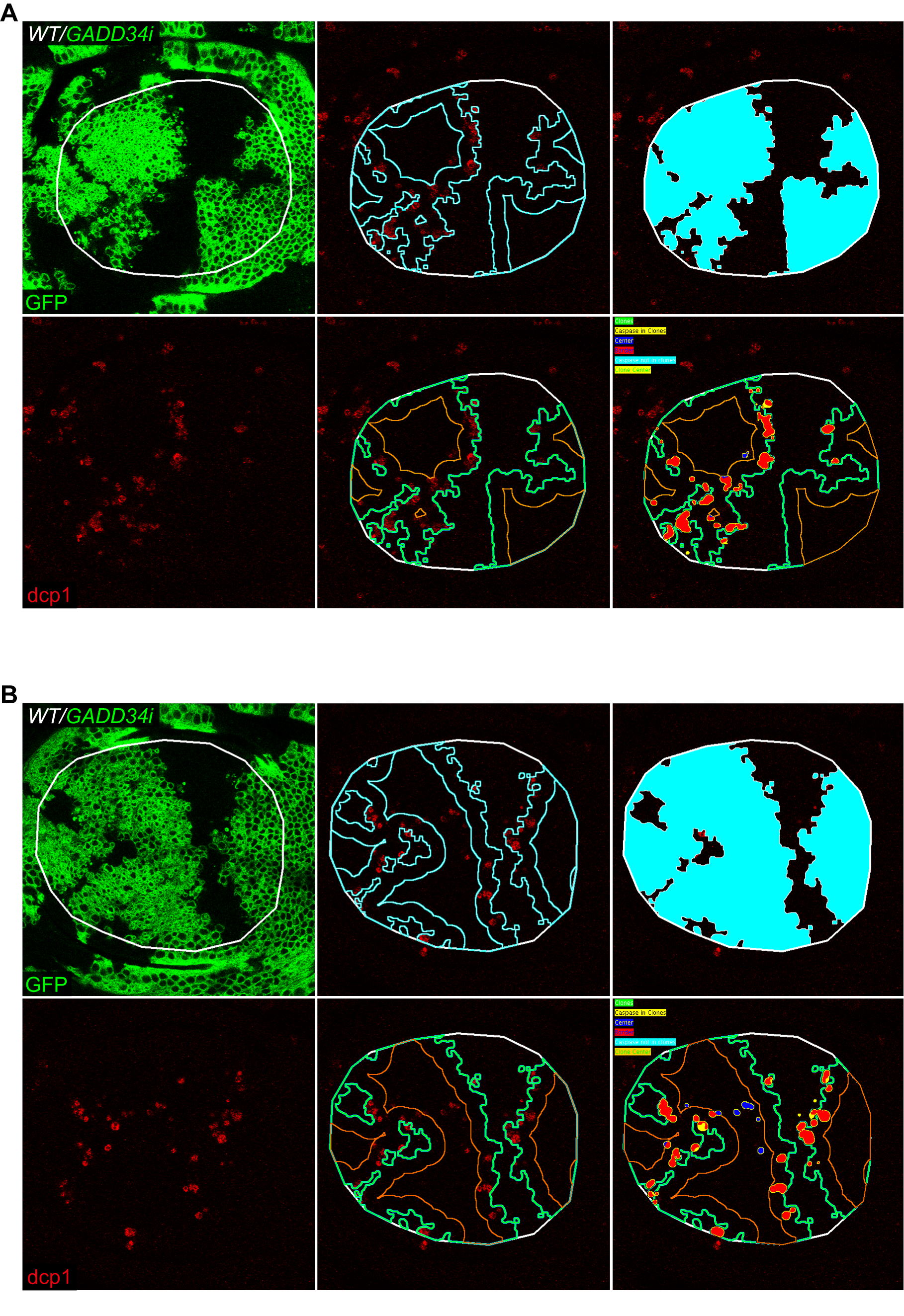

Supplement: S6 Fig — (A-B) Two examples of processed images for a single confocal section from wild-type wing discs harboring cells expressing GADD34-RNAi (GFP positive, top left panels) and immuno-stained for dcp1 (red, bottom left panels). GFP segmentation is shown (cyan, top right panels) and the center and border territories of the GFP patches are defined (top middle panels and bottom middle panels, with center territory indicated with orange lines and border territory indicated with green lines in the bottom middle panels). Segmentation of dcp1 positive cells overlayed with the center and border territories is shown in the bottom right panels: dcp1 positive regions in wild type cells are filled in yellow, border territory dcp1 positive regions are filled in red, and center territory dcp1 positive regions are filled in blue. Single confocal sections are shown here, but the analysis of center and border death was performed across multiple confocal sections, and the density of dying cells presented in Fig 4H is the percentage of the total volume of the border or center territory that is dcp1 positive. (TIF) [file pgen.1009946.s006.tif]

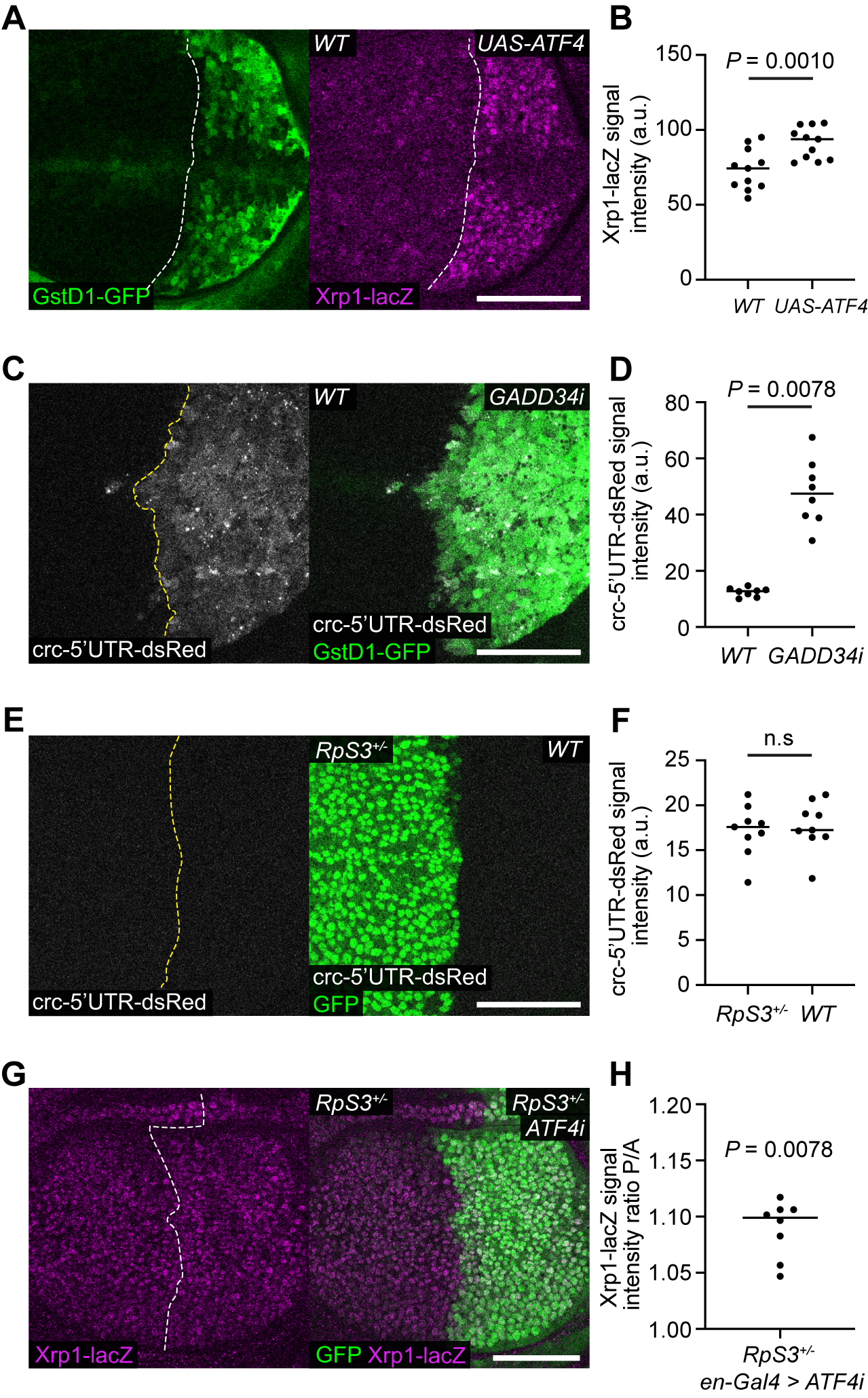

Supplement: S7 Fig — (A-B) A wing disc carrying the xrp1-lacZ reporter and GstD1-GFP (green) and over-expressing ATF4 (UAS-ATF4) in the posterior compartment, immuno-stained with anti-β-galactosidase (magenta) (A), with quantification of xrp1-lacZ signal intensity (B) (n = 11; two-sided Wilcoxon signed-rank test). (C-D) A wing disc carrying an ATF4 translation reporter (crc-5’UTR-dsRed) (grey) and GstD1-GFP (green) and expressing GADD34-RNAi in the posterior compartment (C) with quantification of crc-5’UTR-dsRed signal intensity (D) (n = 8; two-sided Wilcoxon signed-rank test). (E-F) A wing disc carrying crc-5’UTR-dsRed (grey) with an RpS3+/- anterior compartment (GFP positive) and a wild-type posterior compartment (E) with quantification of crc-5’UTR-dsRed signal intensity (F) (n = 9; two-sided Wilcoxon signed-rank test). (G-H) An RpS3+/- wing disc carrying xrp1-lacZ and expressing ATF4-RNAi (ATF4i) in the posterior compartment (GFP positive), immuno-stained with anti-β-galactosidase (magenta) (G), with quantification of the posterior / anterior (P/A) ratio of xrp1-lacZ signal intensity (H) (n = 8; one sample Wilcoxon signed-rank test). (TIF) [file pgen.1009946.s007.tif]
