# Supplementary material for: Xrp1 and Irbp18 trigger a feed-forward loop of proteotoxic stress to induce the loser status
Source: PLoS Genet. 2021 Dec 16;17(12):e1009946. doi: 10.1371/journal.pgen.1009946 (PMC8675655; doi:10.1371/journal.pgen.1009946)
Supplement: Attachment — Submitted filename: Langton_reviewers comments_responses.docx [file pgen.1009946.s009.docx]

Reviewer #1: Summary
This manuscript connects the cell competition associated transcription factors Xrp1 and IRBP18 with proteotoxic stress. The authors show that both factors are needed for the induction of proteotoxic stress response in Minutes. Furthermore, their study reveals that Xrp1 is induced by Nrf2, which is essential for the loser state of Minutes, as well as proteotoxic stress. Combining the results, the authors suggest a feed-forward loop between Xrp1, proteotoxic stress and Nrf2, which ends in the “loser”state of Minutes.

The findings are original, building on previous work in the field and integrates Xrp1 into proteotoxic stress signaling. Consequently, the work will be of interest to many. The presented results largely support the paper’s conclusions however some additional work would be helpful.

We thank reviewer 1 for their positive and helpful comments.

Major points
- Figure 2 shows that ectopic expression of Xrp1 leads to induction of GstD1-GFP, p62 and peIF2α. Given the paper’s focus on both Xrp1 and IRBP18, the authors should also test if overexpression of IRBP18 leads to an increase in p62, p-eIF2α and GstD1. In addition, it would be important to exclude that overexpression per se induces proteotoxic stress (e.g. overexpress GFP or LacZ). The quantification suggests the induction of p-eIF2a and p62 is moderate. Overexpression of a neutral factor, as a control, would make the data more convincing.

Testing the effect of Irbp18 over-expression is a great suggestion. To address this, we acquired a *UAS-Irbp18-HA* line from FlyORF and expressed it in the posterior compartment using exactly the same conditions as for the Xrp1 overexpression experiments, i.e. in the presence of Gal80^ts^ and shifting larvae to the restrictive temperature 24h prior to dissection. Unlike Xrp1 overexpression, Irbp18 overexpression did not induce the proteotoxic stress markers p-eIF2a and p62. The data are included in the new supplementary figure S3. This is consistent with previous data from the Baker lab showing that Xrp1 overexpressing clones, but not Irbp18 overexpressing clones, are eliminated from mosaic tissues.

To address the second part of this major point (whether overexpression of a neutral factor such as GFP would cause proteotoxic stress) we expressed *UAS-GFP* in the posterior compartment with the same conditions as for Xrp1 and Irbp18 expression and examined p-eIF2a and p62 levels (new figure S3). GFP expression did not increase these markers of proteotoxic stress. Levels of p-eIF2a were comparable between compartments and levels of p62 were very mildly but significantly reduced in this experiment.

Overall, these new data show that Irpb18 alone is not sufficient to induce proteotoxic stress and that the upregulation of p-eIF2a and p62 induced by Xrp1 is not the result of overexpression per se.

Regarding the comment that the quantification suggests the induction of p-eIF2a and p62 by Xrp1 is moderate, we thank reviewer 1 for pointing this out. We realised during the revision that there was an error in the image analysis macro that we had used, and this was giving values considerably lower than manual quantification, thus reducing the magnitude of any changes. We have re-quantified every experiment with a new image analysis pipeline (described in our recent preprint Baumgartner at al., Biorxiv) that gives output values that match manual quantifications. The graphs in the figures have been updated accordingly. The reanalysis did not change any of the findings presented in the original submission and in many cases results in a bigger effect size.

If the upregulation of markers still seems moderate, it should be noted that Xrp1 has only been expressed in these discs for 24h to avoid toxicity, therefore it’s possible that stronger induction would be observed with more prolonged Xrp1 expression. We avoided longer periods of expression as this would also cause widespread apoptosis. While the induction of p-eIF2a and p62 in these experiments is moderate, the result is clear, and we have consistently observed a significant increase in these markers upon xrp1 expression (3 biological repeats).

A minor aside related to this, the P values in Figure 2d and f are identical. Is this correct?

The p-values in Figure 2d and Figure 2f (now Figure 3b and Figure 3f) are identical because the Wilcoxon matched pairs signed rank test was used, which compares ranks. As n is the same (GSTGFP and p-eIF2a are in fact quantified from the same discs here) and every disc has higher GST-GFP or p-eIF2a in the Xrp1 expressing posterior compartment than the wild-type anterior compartment, the p-values are the same.

- Figure 4 shows that the induction of proteotoxic stress by the knockdown of GADD34 leads to an increase of peIF2α levels and Xrp1 expression. These results, in combination with the results from Figure 2, are interpreted as feed forward loop. The model illustrated in Figure 5g is predicted from earlier work on the UPR, summarized in reviews such as (Ron and Walter, 2007). For example, ATF4 being upstream of CHOP, a C/EBP protein proposed by Blanco et al 2020 to be the human Xrp1 homolog. A simple testable prediction of the models is the loss of ATF4 should decrease Xrp1 expression. To substantiate the claim, such an experiment should be done. The authors could perform either a knockdown of ATF4 in Minute flies to test if this leads to a reduction of Xrp1 expression. Minimally it should be tested if ATF4 expression is increased in Minutes. This would avoid the technical challenges such as exacerbation of cell competition (as discussed in paper)

We thank reviewer 1 for these suggestions. Our data that ATF4 overexpression induces *Xrp1-lacZ* show that Xrp1 can be activated by ATF4 but do not address whether this mechanism is in place in *RpS3/+* cells. We followed the reviewer’s recommendations and carried out both experiments.

1)We used an ATF4 translational reporter (*crc-5’UTR-dsRed*, which is described in Kang et al. PLOS One, 2005) to test whether ATF4 is increased in *RpS3/+* cells. The reporter was strongly activated in our positive control experiment (by *GADD34-RNAi* expression) but not in *RpS3/+* cells, suggesting that ATF4 is not detectably active in these cells.

2) We knocked down ATF4 in the posterior compartment of *RpS3/+* discs, as suggested and did not see any decrease in *xrp1* expression using *Xrp1-lacZ* as a reporter.

Both experiments can be found in Figure S7. We believe that the simplest explanation for these experiments is that although ATF4 can upregulate Xrp1, it is not involved in Xrp1 upregulation in *RpS3/+* cells. Alternatively, it is also possible that removing ATF4 further stresses Minute cells, as they cannot induce the UPR, leading to Xrp1 upregulation by other pathways. For example, our model predicts that further proteotoxic stress could increase Xrp1 expression via Nrf2. This is now covered in the discussion.

We believe that collectively our experiments support a model whereby Xrp1 upregulation in *RpS3/+* cells is downstream of Nrf2 and of p-eIF2a, yet ATF4 independent. We have shown that p-eIF2a is upregulated in *Rp/+* cells and that increasing p-eIF2a with *GADD34-RNAi* induces *xrp1* expression, so it seems likely that p-eiF2a does contribute to Xrp1 upregulation in *Rp/+* cells. However, this is difficult to test directly, as Minute cells rely on the UPR and p-eiF2a for their survival.

- Given the connection in the paper between proteotoxic stress, cell competition and loser status, it would be great if the authors could show how Xrp1 expression is affected in a Minute situation when proteotoxic stress is alleviated. For example, does GADD34 overexpression block elimination.

We previously expressed *GADD34* in *RpS3/+* clones (in Baumgartner et al, NCB 2021) and it very strongly enhanced *RpS3/+* clone elimination with no clones remaining in wing discs by the time of dissection. Furthermore, others have shown that *GADD34* expression in non-competing Minute discs leads to widespread cell death (Recasens-Alvarez et al, NCB 2021). This is likely because Minute cells need phosphorylated eIF2a to induce the UPR and to slow global translation, both of which help them cope with their underlying proteotoxic stress. Therefore, we are unable to examine *xrp1* expression in *RpS3/+, UAS-GADD34* cells.

Further comments
- In Figure 2b, d, and f the variance of the signal is quite high for the UAS-Xrp1 affected compartment. As noted above the variance and the p-values do not seem to match. To avoid confusion it would help to comment on this and to provide the raw data, including an example of how the quantification was done.

The variance is quite high in the experiments, this is partly accounted for by the variability in the signal intensity from disc to disc (for p62 and peIF2a the WT compartment variance is also quite high). The p-values presented are derived from the Wilcoxon matched pairs signed rank test and are highly significant because the *xrp1* expressing posterior compartment had higher p62 or p-eIF2a or GST-GFP than the anterior compartment in every disc analysed. As requested, we include below a figure for the reviewers, showing macro-output images and pixel intensity values for this experiment. We also provide the raw data for all of the experiments in the manuscript as an Excel file with our revised manuscript.

- The authors speak of RP +/- cells, when they only have used RpS3 +/- genotypes. This could be taken to imply that more RP genes were tested. If only RpS3 was tested this should be They should change this in the text to reduce misunderstandings**.**

Thank you for pointing this out. It is true that while we have provided a role for Xrp1 in three independent routes that induces proteotoxic stress (*RpS3/+*, *mahj-RNAi*, and *GADD34* RNAi) we have only used a single ribosome gene, i.e. *RpS3*. We have gone through the manuscript and used “*RpS3/+*” when we refer to our data on *RpS3/+* cells. When referring to Minute cell competition in general we have used *Rp/+*.

- Figure 3- To faciliate repetition it should be made clearer how the quantification was made. Sufficient would be to provide a marked example – illustrating the clone border and center. In Figure 3j, it is not clear how many clones are present.

We have now included, as a new supplementary figure (Figure S6), two examples of macro-output images for the experiment, which show the clone centre and clone border territories overlayed with the dcp1 signal. Hopefully, this will clarify how the quantification was made.

- Figure 4j vs 4k. it is difficult to reconcile the clear difference in signal seen in 4j with the moderate difference seen in 4k. Again by example it would be helpful clarify how the quantification was done.

Thanks for pointing this out, we have now chosen a disc that is more representative of the increase in *xrp1-lacZ* induced by ATF4 expression; a disc where the Posterior/Anterior (P/A) ratio of *xrp1-lacZ* signal is as close to the median P/A ratio as possible (now Figure S7a-b). Indeed, in every experiment where we compare disc to disc readouts (eg clone size experiments such as in Figure 5a-c and Figure 6a-f, and fluorescence intensity measurements across discs such as in Figure 5d-g and Figure S4b-d) we took care to show representative images that were as close to the median values for the data sets as possible.

- Figure 5g. GADD34 should be added to the proposed model, as it is one of the phosphatases regulating p-eIF2α.

GADD34 is now included in the model.

- ER stress is transduced by several branches in the cell, do the authors predict that also other factors of ER stress play a role in regulating Xrp1 or forming the ‘loser’ state?

This is an interesting question and something we would like to test in future experiments.

- ‘Feed-forward loop’ is a strong description for this mechanism, as it implements an endless cycle of increasing Xrp1 expression, which should lead to death -seen by several studies overexpressing Xrp1 via UAS/Gal4, but surprisingly Minute are viable. Could the authors comment on this?

This is a good point, and it does indicate that additional factors act as inputs into this circuit, so that under pseudo-physiological conditions cells are not inevitably driven to death. This could be in the form of a negative regulator of this circuit that keeps it in check until the stress signal is too strong. Similar mechanisms have been suggested to account for the bistability of ATF4 for example.

- To give their claims more strength the author could also discuss the role of Xrp1 in the toxicity of the ALS-associated FUS orthologue caz mutant phenotype described by Mallik et al.,2018.

Thank you for this important suggestion. The potential importance of our findings linking to Xrp1 to proteotoxic stress are now discussed in the context of proteinopathies, such as ALS.

Reviewer #2: This paper adds nicely to an emerging body of work demonstrating that Rp gene haploinsufficiency leads to severe proteotoxic stress, and activates numerous stress signaling pathways including the ISR. The authors demonstrate mechanistic insight into how the Drosophila transcription factor Xrp1 mediates the intrinsic sensitivity of Rp+/- cells to elimination in mosaic tissues with competing wildtype cells. Several recent studies have identified Xrp1 as a key stress-induced gene that is required with its binding partner, the C/EBP homolog Irbp18, for the majority of phenotypes associated with ribosomal gene haploinsufficiency: the developmental delay, stress pathway activation (DNA damage, JNK, oxidative), cell competition associated gene expression (including expression of Xrp1 itself), and the competitive elimination of Rp+/- cells in mosaic tissues (Baillon 2018, Lee 2018, Ji 2019, Blanco 2020, Recasens-Alvarez 2021). Xrp1 is also induced in response to irradiation in a p53 dependent manner (Brodsky 2004, Akdemir 2007), mediates neurodegenerative toxicity (Gruenewald 2009, Mallik 2018), and is involved in transposon mobilization (Francis 2016), and is thus emerging as a major stress response factor that is induced by numerous different stresses.

Here, the authors follow up on their recent work (Kucinski et al 2017, Baumgartner et al 2021) with a careful set of experiments to investigate how Xrp1 contributes to the Rp+/- phenotype and the “loser status” of these cells in mosaic tissues. Their results lead them to propose that Xrp1 is induced by the transcription factor ATF4 in response to proteotoxic stress, and subsequently functions via a feed forward loop to regulate its own expression and numerous downstream effects that culminate in death of the cells. Overall the work is carefully done and clearly explained, and represents a significant contribution to our understanding of what makes some cells more susceptible to elimination from mosaic tissues.

We thank reviewer 2 for their positive and helpful comments on our manuscript.

Specific comments:
1. In their abstract (line 38) the authors conclude that “Xrp1 induce loser status by promoting proteotoxic stress”. But their data actually suggest a different conclusion: rather, that Xrp1 is induced in response to proteotoxic stress derived from an imbalance in ribosomal stoichiometry in Rp+/- cells, and then functions with Irbp18 to mediate the ISR. They show that loss of xrp1 reduced (“rescued”) p-eIF2a accumulation in Rp+/- cells and also reduced oxidative stress GSTD1-GFP; similar results with Irbp18 RNAi; p62 is also reduced by both. Together, the results suggest that much of the response to proteotoxic stress in Rp/+ cells is mediated by the Xrp1/Irbp18 complex (but is not inducing the stress itself).

Thank you for pointing out that the abstract was missing this important concept. It is indeed true that we show that the initial ribosomal stoichiometry imbalance present in *RpS3/+* cells does not require Xrp1. We agree that p-eIF2a and GSTGFP rescue results could be interpreted as Xrp1 knockdown rescuing the response to proteotoxic stress rather than the stress itself. However, the rescue of p62 labelled aggregates by *xrp1-* or *irbp18-RNAi*, and the rescue of LSU accumulation by removal of one copy of *xrp1* suggest that proteotoxic stress is also being rescued. These are amongst the main arguments that lead us to suggest that Xrp1 is involved in a feed forward loop both upstream and downstream of proteotoxic stress in *Minute* cells. We have modified the abstract to reflect better this overall message.

2. The authors show that Xrp1 is required to eliminate Rp+/- cells in mosaics, as shown previously by other labs, and that cell elimination by competition between WT and mahj-RNAi expressing cells is also reduced by knockdown of Xrp1. They conclude that a common mechanism is at work in elimination of losers. This conclusion would be significantly stronger if they could show that loss of Xrp1 also prevented p-eIF2a in the mahj knockdown cells.

We thank reviewer 2 for suggesting this experiment. We addressed this by comparing the level of p-eIF2a induction in *mahj-RNAi* cells versus *mahj-RNAi* + *xrp1-RNAi* cells. We used a slightly longer heat-shock than for the previous *mahj-RNAi* clone size experiments to increase the size of the *mahj-RNAi* patches. We are pleased to report that *xrp1* knockdown significantly reduces p-eIF2a accumulation in *mahj* deficient cells, and this was seen with three biological repeats. This new data is included in the new Figure 2 and suggest that *xrp1* removal rescues proteotoxic stress in two different loser backgrounds, *mahj* and *RpS3/+*.

3. It is interesting that Irpb18 knockdown is not as efficient at preventing loser cell elimination as loss of Xrp1 (Fig. S1). Do the effects of overexpression of Xrp1 require Irbp18?

Irbp18 knockdown is indeed less efficient than Xrp1 knockdown at rescuing cell elimination. This may simply reflect differences in the efficacy of the RNAi lines used. To address whether the effects of Xrp1 overexpression require Irbp18, we generated a new recombinant chromosome – *en-Gal4, UAS-GFP, UAS-irbp18-RNAi*. We were concerned that the short burst of Xrp1 used for the Figure 2 (now Figure 3) overexpression experiments (24h at the Gal80^ts^ restrictive temperature) would too short for effective RNAi knockdown of Irbp18. Therefore, we checked whether 24h of *Irbp18-RNAi* expression would be enough to affect normal *xrp1* expression using *Xrp1-lacZ* as a readout. We found that shifting *tub-Gal80ts / +, en-Gal4, UAS-GFP, UAS-irbp18-RNAi / +, xrp1-lacZ / +* larvae to the restrictive temperature 24h prior to dissection was sufficient to reduce levels of *Xrp1-lacZ* suggesting that knockdown of Irbp18 had occurred over this time window. We then compared the level of p62 induction in *UAS-Xrp1* versus *UAS-Xrp1* + *irbp18-RNAi* compartments. We are pleased to report that Irbp18 does seem to be (at least partially) required for Xrp1 to induce p62, which is consistent with data from the Baker lab, showing that elimination of Xrp1 clones is partially rescued in *Irbp18* mutant discs. This new data is shown in Figure S4.

4. Based on their data that expression of ATF4 induces xrp1-lacZ and the literature, they suggest ATF4 is involved in initial Xrp1 upregulation. It would be nice to see this, however: If the authors block cell death in the Rp+/- cells (e.g., UAS-p35 or UAS-miRHG) would they be able to see that ATF4 is required for Xrp1 induction?

This is an important question that is addressed directly in the revised manuscript

1)We used an ATF4 translational reporter (*crc-5’UTR-dsRed*, which is described in Kang et al. PLOS One, 2005) to test whether ATF4 is increased in *RpS3/+* cells. The reporter was strongly activated in our positive control experiment (by *GADD34-RNAi* expression) but not in *RpS3/+* cells, suggesting that ATF4 is not detectably active in these cells.

2) We knocked down ATF4 in the posterior compartment of *RpS3/+* discs, as suggested and did not see any decrease in *xrp1* expression using *Xrp1-lacZ* as a reporter. Blocking cell death was not necessary for the experiment and would have had the complication of inducing tumours as recently shown by Recasens-Alvarez et al NCB 2021.

On the basis of this new data, which can be found in the new Figure S7, we believe that the simplest explanation for our experiments collectively is that although ATF4 can upregulate Xrp1, it is not involved in Xrp1 upregulation in *RpS3/+* cells. This is discussed in detail in the manuscript.

5. line 293: “this suggests that in Rp/+ tissues, proteotoxic stress activates Xrp1 by two routes, one via the UPR and ATF4, and the other via Nrf2.” I agree this makes sense, but this seems like a strong conclusion given that the data used to support it (Fig. 5) are from wildtype discs in which they express UAS-xrpr1-RNAi in UAS-Nrf2 overexpressing clones; could the conclusion be made clear that it is only one possibility?

Our revised discussion regarding the regulation of *xrp1* transcription by the UPR and Nrf2 makes it clear that this is hypothetical and we suggest there may be additional inputs to the *xrp1* promoter in *RpS3/+* cells.

6. Lines 297-299: “…proteotoxic stress and the Xrp1/Irbp18 complex, which is required for the elimination Rp+/- cells in competing mosaic tissues”. However, all of the markers of “loser status” used here (GstD1, p-JNK, Xrp1, p-eIF2a, p62, etc) are also required for the cell autonomous “Minute condition”; it should also be made clear here that these effects are intrinsic to the mutant, and are not restricted to cell competition in mosaics.

Thank you. We have amended this accordingly.

7. In the Fig. 5g model, where the Rp+/- condition/ Rp stoichiometric imbalance is proposed to lead to NRF2 induction, which then leads to Xrp1 induction. Interesting ideas, but speculation at this point – do they know that ATF4 is not required for Nrf2/GSTD induction? Also, the two dotted arrows to loser status make it more clear that this is speculative, but text seems to point to more linear relationships (i.e., Lines 293-294).

It is true that we cannot rule out that the UPR is upstream of Nrf2 activation, however at this stage we have no direct data to support it being directly downstream of the UPR and ATF4. Conversely, at least in mammals, there are two mechanisms that are known to activate Nrf2 directly from proteotoxic stress: the p-eIF2a kinase PERK, which leads to eIF2a phosphorylation downstream of proteotoxic stress is also a kinase for Nrf2, suggesting a direct mechanism of activation; in addition, proteotoxic stress can also inhibit the proteasomal degradation of Nrf2, induced by the E3 ligase KEAP1, providing another mechanism for direct proteotoxic stress-induced Nrf2 activation that is p-eIF2a and ATF4 independent.

In light of this, it seemed less speculative to place Nrf2 directly downstream of proteotoxic stress than to place it downstream of p-eIF2a. We have however added a sentence in the discussion to suggest that it might also be possible that p-eIF2a directly induces Nrf2 activation.

8. The authors propose that the initial proteotoxic stress comes from an imbalance between SSU and LSU ribosomal proteins (see note below, they make it seem here like it’s a new observation). Then, proteotoxic stress in Rp+/- cells induces Xrp1 expression. The Baker lab has provided evidence that RpS12 is specifically required for Xrp1 induction, which may be related to the SSU/LSU imbalance. Could the authors speculate about the role of RpS12 in the SSU/LSU imbalance and activation of Xrp1? The Baker lab has proposed that RpS12 triggers Xrp1 expression, perhaps these ideas are related. It would be appropriate to cite the previous work and speculate on links to their own interpretations.

We have added a sentence in the discussion about RpS12 and its regulation of Xrp1 transcription in Minutes.

We have also rephrased the sentence on the imbalance between SSU and LSU. We now emphasize that the novel finding is that such imbalance is at least in part Xrp1-independent.

9. Overall the authors have a tendency to over interpret and/or overstate their conclusions and sometimes forget to cite previous work showing the similar results. Examples: in lines 299-307: this paragraph describes their speculative model, but is written as if it was conclusive. Qualifying this description with “our data suggest a model in which…..” could mitigate this problem. Also, some of the statements made here are not exclusive to this current work, but seem implied to be: e.g. lines 299-300: this has also been shown by others (Lee and Baker, Baumgartner, Recasens-Alvarez, etc), so the authors should give them credit as well. The authors should also give more credit to Baillon et al 2018, who did chIP seq after over-expression of Xrp1 in wing discs and found that Xrp1 binds to its own locus and upregulates it, as well as many other genes associated with cell competition, including puc, hid and rpr. They also reported the homology with CEBP and postulated on its role.

In the revised manuscript we have been more careful not to over interpret our results, including in the discussion where we describe our working model.

It is true that the SSU/LSU imbalance has been shown in previous papers (Baumgartner et al and Recasens Alvarez et al). The novel finding here is that an SSU/LSU imbalance exists even when Xrp1 is removed. To make this clear we have changed the sentence at the start of the discussion accordingly.

We now reference Baillon et al for discovering that Xrp1 regulates its own expression (at the start of the results) and expression of proapoptotic genes (in the results section describing our Xrp1 overexpression experiments), and for showing that Xrp1 overexpression induces high levels of cell death (in the intro and discussion). We also cite appropriately Baillon et al and Blanco et al on the evidence suggesting that Xrp1 may be functionally equivalent to CHOP (in the results section).

10. Line 212: “If Xrp1 and Irbp18 are required in competition because they induce proteotoxic stress”, add “cell” in front of competition.

Thank you. This has been corrected

11. Cell death at clone borders vs centers: I always find this difficult to assess – in Fig. 3J, for example, the caspase stain appears to overlap with nuclei (in the same focal plane?) – whereas dying cells are primarily found to delaminate and fragment (as they show in FigS2e). If they do a 3D reconstruction of several sections (apical to basal), do they also see these delaminated caspase-marked cells? What is their distribution?

We have now added a supplementary figure (Figure S6) that shows two examples of output images from the macro we use for analysis of cell death within clones. The border and centre territories are shown, overlayed with the dcp1 signal, to clearly show where the dying cells are within the clones. S6a is exactly the same disc and confocal plane as shown in (now) Figure 4j so readers can see where the dying cells are relative to the clone borders and centres. These are individual planes; however, the analysis is carried out across the wing disc, with the exception of the most basal planes as the GFP starts to fade in parts of the clones, and it becomes difficult to identify and segment clone boundaries.

*GADD34-RNAi* expressing clones with the conditions used for this experiment (shown in the schematic in the new Figure S5f) have dcp1 positive cells within the plane of the nuclei, and the number of dcp1 positive cells increases considerably towards the basal side of the disc, as the reviewer mentions is expected. This is true for all competition experiments. In this specific experiment, Gal80ts is present and clones have only been expressing *GADD34-RNAi* for 24h, which is why the clones (and many dcp1 positive cells) are still in the plane of the epithelium. In the experiments performed in the absence of Gal80ts (now shown in Figure S4d-e and Figure 5a) the clones have been expressing *GADD34-RNAi* for 3 days and are absent or only remain as basally extruded fragments in some discs.

12. Fig. S2: The title to this figure is “GADD34 knockdown induces the loser status”. The authors find that Gadd34-RNAi induces p-eIF2a, pJNK and cell death, and go on to state that “increased border death is a hallmark of cell competition”, citing the Baker lab. Hallmark is a tricky word as it can imply an absolute. However, in other contexts of cell competition death is not primarily at clone borders, nor is it always at clone borders even in Rp+/- induced cell competition. To be more broadly accurate about “the loser status” (and perhaps more appropriately circumspect) I suggest that the authors change the title to something like “GADD34 knockdown phenocopies Rp+/- loser status”, or “GADD34 knockdown induces traits common to Rp+/- loser cells”.

We are not sure why the reviewer states that death is not always at clone border in Minute competition. Not only the Baker lab but our lab has shown extensively that while there is some death in Minute cells, competition induced death is exquisitely at clone borders (see Baumgartner et al NCB 2021 and also Baumgartner et al., BioRx doi: <https://doi.org/10.1101/2021.07.06.451317>).

We have nevertheless changed the figure title to “GADD34-RNAi cells induce JNK signalling and are eliminated from mosaic tissues.”

13. On line 259, the authors state that titration of Gal4 by multiple UAS transgenes was controlled for in experiments using UAS-Gadd34-RNAi plus/minus UAS-xrp1-RNAi by adding “an inert” transgene to controls. I have two thoughts to consider here. First, this control RNAi from the VDRC does not appear lead to production of any RNA, making it formally possible that Pol II and associated factors are not recruited/assembled appropriately – is this really a good control for Gal4 titration, over say UAS-GFP or UAS-lacZ? Second, the authors indicate that UAS transgenes were controlled for in all subsequent experiments, which is very good (although of course should be standard practice when using multiple UASes). However, some experiments have even >2 UAS transgenes: e.g., Fig. 4b, 5a, d, e – were these controlled for as well?

All experiments in the original submission comparing the effect of one UAS to the effect of two UASes were controlled for using the VDRC *60101* line (aka *40D^UAS^*). For example, in Figure 4a (now Figure 5a) there are three UASes (*UAS-GFP*, *40D^UAS^*, and *UAS-gadd34-RNAi*) and the discs we are compare these to, in Figure 4b (now Figure 5b), also have three UASes (*UAS-GFP*, *UAS-xrp1-RNAi*, and *UAS-GADD34-RNAi*). All experiments in Figure 5 (now Figure 6) are controlled for this way and we have indicated the full genotypes for all experiments in the methods.

The only experiment that does not control for multiple UASes this way is the new experiment done for the revision to address whether the effects of Xrp1 require Irbp18 (reviewer 2, point 3 – Figure S4) because the genetics did not easily allow for the *60101/40D^UAS^* chromosome to be included. We do not think this diminishes the result as, after all, Gal4 titration is only a theoretical possibility.

Regarding whether using the *40D^UAS^* is a good control to use, we think it is likely that pol II and associated factors would be recruited to this site as Vissers et al (Nature Comms, 2016) showed that the nearby *tiptop* gene was upregulated ~2 fold in *GMR-Gal4, 40D^UAS^* eye discs. Therefore, we believe this is a good control for Gal4 titration.

Given the mild upregulation of tiptop, in the revision we have checked whether *40D^UAS^* in combination with a strong Gal4 driver (*hh-Gal4*) would affect loser status readouts when expressed in either wild-type or *RpS3/+* cells. Our data confirm that *40D^UAS^* does not itself affect those markers (Figure S2)

Using *UAS-GFP* or *UAS-lacZ* would’ve been another viable alternative. Therefore, we have repeated one of the key experiments using *UAS-GFP* as the control UAS. We found that *UAS-xrp1-RNAi + UAS-gadd34-RNAi* cells still had lower levels of p-eIF2a than *UAS-GFP + gadd34-RNAi* cells. This is included below as a figure for reviewer’s assessment.

14. The interesting statement is made that (line 309) “ATF4 plays a dual role: it promotes Xrp1 expression in Rp+/- cells, possibly along with Irbp18; however, it is also required for expression of chaperones..”. Is there any evidence from their RNA seq data (Kuscinski 2017) or other published transcriptome data (Baillon et al 2019, Boulan et al 2019, Baker lab) that suggest that Xrp1 is required for chaperone gene expression?

This sentence has now been removed from the discussion as the new data suggests that ATF4 does not regulate Xrp1 expression in *Rp/+* cells, but this is an interesting question.
